# An Overview of Cellular and Molecular Determinants Regulating Chemoresistance in Pleural Mesothelioma

**DOI:** 10.3390/cancers17060979

**Published:** 2025-03-14

**Authors:** Lourdes Cortes-Dericks, Domenico Galetta

**Affiliations:** 1 Department of Biology, University of Hamburg, 20146 Hamburg, Germany; 2 Division of Thoracic Surgery, San Giovanni Bosco Hospital, 10154 Turin, Italy; domenico.galetta@aslcittaditorino.it

**Keywords:** malignant pleural mesothelioma, chemoresistance, drug resistance mechanisms, drug-resistant cancer stem cells

## Abstract

Malignant pleural mesothelioma (PM) is a highly aggressive disease of the lung pleura associated with poor prognosis. Despite advances in improving the clinical management of this malignancy, there is no effective chemotherapy for refractory or relapsing PM. The acquisition of resistance to standard and targeted therapy in this disease is a foremost concern; therefore, a deeper understanding of the complex factors surrounding the emergence of drug resistance is deemed necessary. In this review, we will present broad insights into various cellular and molecular concepts, accounting for the recalcitrance of PM to chemotherapy, including signaling networks regulating drug tolerance, drug resistance-associated proteins, genes, and miRNAs, as well as the critical role of cancer stem cells. Identification of the biological determinants and their associated mechanisms may provide a framework for the development of appropriate treatment.

## Simple Summary

Pleural mesothelioma (PM) is an aggressive malignancy of the lung strongly associated with asbestos exposure. It is characterized by a prolonged latency period of 20–40 years, leading to delayed detection and poor prognosis. The median survival of PM patients is only 8–14 months after diagnosis. Despite significant advancements in diagnosis and treatment, therapeutic options remain limited, primarily consisting of platinum-based chemotherapy. The emergence of resistance to chemotherapeutics poses a major challenge in improving overall survival of patients. In this review, we discuss the various cellular and molecular determinants of drug resistance in PM. Understanding these factors may facilitate the identification of novel therapeutic targets and support the development of innovative drugs designed in response to known resistance mechanisms.

## 1. Introduction

Pleural mesothelioma (PM), a rare and aggressive malignancy arising in the mesothelial cells of the pleural surface, is significantly associated with a dismal prognosis [1,2], with a median overall survival of between 8 and 14 months [3]. Occupational asbestos exposure for more than 40 years is a predominant etiological factor [4,5]. The percentage of non-asbestos-associated PM is generally estimated at 10–20%, caused by predisposing factors such as SV-40 viruses, recurrent infection, or genetic disposition [6]. The immune system has also been implicated in the pathogenesis and response of PM to therapy [7,8,9]. Consequently, antineoplastic agents, such as immune checkpoint inhibitors (ICIs), and targeted agents have recently been evaluated in this malignancy [10]. Histologically, PM is classified into epitheloid, sarcomatoid, or biphasic types, each having its own clinical course and prognosis [11,12]. Among them, sarcomatoid mesothelioma is the most aggressive form and has the worst prognosis [13].

The combination of platinum-based drugs and pemetrexed remains the mainstay of mesothelioma treatment, and aggressive surgery may be considered for selected patients presenting with early disease [14]. However, most patients presenting with advanced disease will receive palliative systemic therapy [11]. The conventional treatment modalities for PM include surgery (extrapleural pneumonectomy or, more recently, pleurectomy/decortication) for resectable cases, often combined with chemotherapy (CT) and/or radiotherapy (RT), and CT or RT for unresectable tumors [14]. Despite advances in understanding PM pathobiology, including modest alleviation of disease-related symptoms, the management of PM is still far from ideal. Mortality is high, most likely due to late diagnosis and drug resistance [15].

In PM, first-line treatment consisting of cisplatin and pemetrexed does not achieve optimal outcomes, resulting in an overall survival of ~13 months [16,17]. Double immunotherapy combining nivolumab and ipilimumab has recently emerged for frontline or second-line therapy, with an overall survival (OS) benefit that was mainly observed in patients with the non-epithelioid histological subtype [18,19]. The cytotoxic effect of cisplatin (CDDP), a platinum-based chemotherapeutic drug, is through the formation of DNA–platinum adducts, followed by the DNA damage response [20,21]. Pemetrexed (PMX), an antifolate, arrests DNA and RNA synthesis by inhibiting thymidylate synthase, dihydrofolate reductase, and glycinamide ribonucleotide formyltransferase that are necessary for purine and pyrimidine synthesis [22,23].

As with other cancer types, 90% of drug tolerance to chemotherapeutics develops during treatment, either intrinsically or acquired. Intrinsic resistance occurs before drug treatment, while acquired resistance results after drug uptake, with each accounting for approximately 50% of chemoresistant patients [24]. Intrinsic resistance may be due to the pre-existing inherent genetic mutations of a tumor, activation of signaling pathways as a defense mechanism against anticancer drugs, the heterogeneity of tumor cell populations, including the drug-tolerant cancer stem cell fraction, and pharmacological factors such as inadequate intracellular drug concentration at the tumor site [25]. Acquired resistance may be caused by the activation of a second proto-oncogene, subsequently serving as the driver gene, altered mutations or expression profiles of the drug targets, and alterations in the tumor microenvironment after drug therapy [24].

According to past and current research, the mechanisms of drug tolerance in different types of cancer involve the presence of one or a combination of oncogenes, transporter pumps, increased DNA repair, autophagy, epithelial–mesenchymal transition (EMT), metabolic reprogramming, cancer stemness, and cancer-derived exosomes, among others [26,27,28,29,30,31].

Herein, we present a broad review of the cellular and molecular determinants of chemoresistance in PM, representing one of the major difficulties in the treatment of this disease.

## 2. Determinants of Drug Resistance in PM

### 2.1. Signaling Networks Regulating Drug Resistance

Advances in molecular/cellular-based assays and techniques enable current studies to identify and characterize the biological determinants and related complex networks driving cancer cells’ ability to circumvent the toxic effects induced by chemotherapeutic treatment.

PM is predominantly associated with chronic inflammation resulting from asbestos exposure [32]. In this process, the activation of nuclear factor kappa-light-chain-enhancer of activated B cells (NFκB) and signal transducer and activator of transcription 3 (STAT3) plays a critical role in mediating refractoriness to chemotherapeutics and promoting the mesenchymal attributes of tumor cells. According to Cioce and colleagues [33], butein (3,4,2′,4′-tetrahydroxychalcone), a naturally occurring NFκB/STAT3 inhibitor can restrain pSTAT3 phosphorylation, the nuclear localization of NFκB, and NFκB/STAT3 interactions. This inhibition correlates with the suppression of genes involved in cancer progression, and the production of proangiogenic cytokines such as VEGF, IL-6, and IL-8, which are essential growth factors for PM.

PM cells go through accelerated senescence after PMX treatment, characterized by a senescence-associated secretory phenotype (SASP). The results of a previous study [34] affirmed that conditioned media from these senescent PM cells can induce epithelial-to-mesenchymal (EMT)-like clonogenic and chemoresistant cell fractions, marked by high aldehyde dehydrogenase (ALDH) activity. Mechanistic analysis revealed that the oncoprotein RAS(v12) induces SASP-like changes in untransformed mesothelial cells and identified STAT3 as a key downstream effector of SASP signaling. RNA ablation of STAT 3 reduced the expression of EMT genes and the number of SASP-mediated ALDH^bright^ cells, resulting in an antitumorigenic effect. The same group found additional molecular events that regulate ALDH1A3 expression in the drug-resistant cell population in PM. STAT3 (tyr-705)-NFkB (p65)-dependent suppression of DNA Damage Inducible Transcript 3 (DDIT3) expression ensures high levels of CCAAT-enhancer-binding protein (CEBP) β-dependent ALDH1A3, which influences the survival and drug tolerance of ALDH+ cells to PMX and cisplatin, demonstrating a DDIT3-specific mechanism of drug resistance. [35].

Chemotherapy drives endoplasmic reticulum (ER) stress, which in turn activates the C/EBP β liver-enriched inhibitory protein (LIP), a pro-apoptotic and chemosensitizing factor. In PM cells derived from poorly cisplatin-responsive patients, LIP was found to be degraded by constitutive ubiquitination, whereas its overexpression recovered cisplatin’s pro-apoptotic effect. This described effect occurred via activation of the C/EBP homologous protein (CHOP)/tribbles-related protein 3 (TRB3)/caspase 3 axis and the upregulation of calreticulin, which triggered PM cell phagocytosis by dendritic cells and increased the abundance of anti-tumor CD8^+^/CD107^+^ cytotoxic lymphocytes. Functionally, the loss of LIP in PM samples mediated cisplatin resistance, rendering this as an indicator of cisplatin response in PM [36].

Increased transcript expression of macrophage colony-stimulating factor-1 receptor (M-CSF/CSF-1R) in PM specimens relative to normal tissues suggests that CSF-1R could be used to identify a chemoresistant cell population in PM primary cultures and cell lines. Single-cell characterization demonstrated that CSF-1R-positive cells manifested attributes of pluripotency, EMT transition, and detoxifying factors, indicating that they represent clonogenic, chemoresistant, precursor-like cell fractions. Activating CSF-1R in untransformed mesothelial cells was sufficient to convey clonogenity and PMX resistance. The survival of CSF-1R positive cells depended on active v-akt murine thymoma viral oncogene homolog 1 (AKT) signaling, which generated increased levels of transcriptionally competent β-catenin. Inhibiting AKT lowered the activity of β-catenin-dependent reporters, thereby sensitizing the cells to senescence-induced clonogenic death following PMX treatment [37].

The epidermal growth factor receptor (EGFR), a membrane-bound receptor tyrosine kinase (RTK), is mostly deregulated by somatic mutations and amplification and is overexpressed in different cancers such as non-small cell lung cancer, esophageal cancer, and PM [38,39]. Xin and coworkers [40] provided evidence that more than 50% of EGFR-positive esophageal cancer (EsC) and PM cell lines were resistant to EGFR–tyrosine kinase inhibitors (TKIs). Susceptibility to EGFR-TKI growth inhibitory action correlated positively with the expression of the epithelial gene marker E-cadherin but negatively with mesenchymal gene markers. The acquired resistance to EGFR-TKI in intrinsic sensitive cancer cells correlated with spontaneous loss of E-cadherin, whereas the ectopic expression of E-cadherin sensitized drug-resistant cells to EGFR-TKI, accounting for a direct implication of E-cadherin in ensuing EGFR-TKI resistance. Considering that low expression or the complete absence of E-cadherin is one of the hallmarks of EMT, the observed resistance of PM cells to EGFR-TKI could have been effected in part by E-cadherin involving EMT.

One of the major challenges in cancer therapy is understanding why tumors resist apoptosis, which is crucial for either eliminating malignant cells or making them more susceptible to conventional treatments [41]. Barbone and colleagues [42] found that mesothelioma-derived spheroids acquired resistance to different apoptotic agents such as the tumor necrosis factor-related apoptosis-inducing ligand (TRAIL), ribotoxic stressors, histone deacetylase, and proteasome inhibitors, which are otherwise highly effective against PM when grown as a monolayer. This acquired resistance was inhibited by rapamycin, an inhibitor of the phosphatidylinositol 3-kinase/Akt/mammalian target of the rapamycin (mTOR) pathway, suggesting involvement of mTOR. Furthermore, silencing S6K, a downstream target of mTOR, generated the same effect as rapamycin, further supporting the role of mTOR and S6K in the acquired apoptotic resistance in PM spheroids. Intriguingly, the sensitivity of the tumor spheroids to GDC-0980, a PI3K//mTOR kinase inhibitor, was not linked to the Akt/mTOR pathway but was instead associated with the presence of ATG 13 puncta, a marker of early autophagy [43].

Hippo signaling plays a crucial role in modulating cell proliferation and the progression of various diseases, including cancer, and is believed to play a pivotal role in the development of chemoresistance [44], particularly Hippo-yes-associated protein (YAP)/transcriptional coactivator with PDZ-binding motif (TAZ) signaling in several solid cancers. Deregulation of the Hippo pathway involving YAP/TAZ activation has been linked to resistance to targeted therapies, chemotherapies, radiation, and, most likely, immunotherapies through diverse mechanisms. Despite various complex mechanisms, they converge on YAP/TAZ-triggered transcription either via inactivation of YAP/TAZ-negative regulators or by direct activation of YAP and/or TAZ [45]. For instance, Song et al. [46] found that upregulated expression of YAP in A549 lung cancer-derived tumor spheres was associated with enhanced CDDP resistance and that silencing of YAP enhanced the cytotoxic effect of CDDP by increasing cell death. In PM, the overexpression of YAP/TAZ has been linked to oncotherapy resistance, resulting in lower efficacy of available treatment modalities. On account of this, YAP/TAZ/TEAD signaling has been presented as a novel therapeutic target in PM [45,47].

Fibroblast growth factor (FGF)/FGF receptor (FGFR) signaling plays a crucial role in cancer progression and tumor maintenance [48]. Fibroblast growth factor 5 (FGF5), a member of the FGF family, has been implicated in promoting therapy resistance by activating FGFR signaling pathways [49]. In both PM cell lines and tissue specimens, Grusch et al. [50] found that high FGF5 expression correlated with cisplatin resistance and poorer overall survival (OS), which appeared to be linked to cisplatin resistance. Interestingly, high FGF5 expression sensitized PM cells to FGFR inhibitors, highlighting its therapeutic potential. Despite the observed correlation between high FGF5 expression and cisplatin resistance, the study did not identify a specific FGF-dependent resistance mechanism.

### 2.2. Drug Resistance Regulation by Proteins and Genes

The interaction between anticancer drugs and aberrantly generated genes or proteins can change the therapeutic attributes of the former and allow cancer cells to escape chemotherapeutic treatment.

Transforming growth factor alpha (TGFα) is one of the seven ligands of the epidermal growth factor receptor (EGFR or HER1). Its role in promoting resistance to valproic acid (VPA) and doxorubicin was reported recently in two PM cell lines. In this study, TGFα was detected as a major component in resisting drug treatments such that overexpression of TGFα promoted drug tolerance whereas silencing correlated with a higher degree of apoptosis. Further data revealed that multi-targeted inhibition of EGFR improved response to VPA and doxorubicin in vitro and reduced tumor growth in the PM mouse model. Finally yet importantly, TGFα but not EGFR expression correlated with patient survival, suggesting that TGFα is a key factor in resistance to PM chemotherapeutics [51].

Metallothionins (MT) are sulfhydryl-rich proteins, with antioxidant properties [52], involved in different biological processes such as cell proliferation and apoptosis [53]. In PM, the roles of MT1A, 1B, and 2A were investigated for their contributions to resistance against platinum-based treatments. Knockdown studies showed that low levels of MT2A expression led to increased apoptosis during cisplatin treatment in three PM cell lines. Consistently, silencing of MT2A in MSTO-21H cells demonstrated that targeting MT-driven resistance mechanisms can improve the response to cisplatin-based therapy in vitro [54]. A separate study identified a negative correlation between MT expression and OS and progression-free survival in PM patients, denoting resistance to platinum-based therapy, particularly in progressive PM patients with upregulated levels of MT. This drug resistance was attributed to promoter DNA hypomethylation and expression of miRNA-566, a direct regulator of the copper transporter SLC31A1 and putative regulator of MT1A and MT2A [55].

Thymidylate synthase (TYMS) is the key enzyme in the synthesis of thymidylate, an essential precursor for DNA biosynthesis [56]. Overexpression of TYMS, often due to gene amplification in cancer cell lines, is strongly linked with resistance to 5-FU (5-fluoacil) [57]. In PMX-resistant cell lines, *TYMS* overexpression was detected as compared to parental cells, leading to resistance to PMX. This acquired drug resistance might be due to the prevention of deoxyuridine monophosphate (dUMP) accumulation, and the increased formation of deoxythymidine monophosphate (dTMP) pools resulting from higher TYMS levels [58]. Intriguingly, another study found that low TYMS expression does not always correlate with PMX sensitivity as increased expression was observed in only two out of four PM cell lines, suggesting that additional mechanisms contribute to both natural and acquired resistance to PMX in mesothelioma [59].

More recently, the role of arachidonic acid (AA), an integral constituent of the biological cell membrane, providing fluidity and flexibility [60], was investigated in promoting the drug tolerance of PM to PMX. In vitro studies revealed that the early cytosolic phospholipase A2 (cPLA2)-dependent release of AA from PMX-treated PM cells activated NFκB, which promoted gene expression and cell subpopulation rearrangements consistent with a chemoresistant phenotype. These data suggest that AA as an early initiator of the adaptive response to PMX in drug-resistant PM [61].

The functional role of proton-coupled folate transporter (PCFT), crucial for folate and antifolate transport [62], was explored relative to the sensitivity of mesothelioma cells and PM spheroids to PMX as well as its correlation with overall survival (OS) and disease control in PMX-treated PM patients. In the present study, MSTO-211H cells, which express the highest levels of PCFT, were the most sensitive to PMX, whereas H28 cells, with the lowest PCFT mRNA and protein levels, showed the highest resistance to PMX. Accordingly, patients with low PCFT levels had significantly lower rates of OS in both test and validation cohorts, suggesting that targeting PCFT-promoter methylation could eliminate PMX-resistant cells, in patients with low PCFT levels [63]. Another study identified a hypoxia/PCFT-dependent mechanism of chemoresistance to PMX. In PM specimens, decreased PCFT levels were associated with hypoxia, as indicated by high levels of the hypoxic marker carbonic anhydrase IX (CAIX). Overexpression of CAIX correlated with low PCFT expression and reduced sensitivity to PMX. At the cellular level, hypoxic and PCFT-silenced cells exhibited upregulated lactate dehydrogenase A (LDH-A), which correlated with shorter patient survival in PM and diffuse peritoneal mesothelioma (DPM) patients [64].

BRCA 1-associated protein 1 (*BAP1*), a tumor suppressor gene located on chromosome 3p21.1 [65], encodes BAP1, now widely recognized as a deubiquitinating enzyme (DUB). Germline mutations of BAP1 elicit augmented susceptibility to tumor development in several cancers including PM [66]. A retrospective biomarker study of PM patients and PM cell lines with or without the BAP1 protein reported the essential role of BAP1 relative to drug tolerance to cisplatin. In this work, somatic *BAP1* mutations occurred in the early phase of PM disease, indicating that most patients do not acquire drug resistance during treatment but that it is instead caused by intrinsic resistance. In vitro data showed that alterations in *BAP1* led to resistance to cisplatin, most likely due to the loss of the BAP1 protein, leading to a diminished rate of apoptosis through transcriptional downregulation of apoptotic genes. This effect was possibly modulated by the interplay between BAP1, Host cell factor 1 (HCF1), and E2F transcription factor 1 (E2F1) [67]. Similarly, in a separate study, BAP1 inactivation in PM cells conveyed resistance to gemcitabine, supporting the notion that BAP1 status has a potential role in predicting the sensitivity of PM cells to gemcitabine [68].

E-cadherin is conventionally recognized as a tumor suppressor because of its pivotal role in preserving epithelial cell–cell adhesion, thus suppressing epithelial–mesenchymal transition (MET), a crucial factor in cancer development and metastasis [69,70]. However, in the study by Kato et al., E-cadherin exhibited a tumor-promoting effect. These authors provided evidence that a subset of Merlin-deficient PM cell lines were resistant to the FAK inhibitor VS-4718. Molecular-based analyses revealed a correlation between E-cadherin mRNA levels and VS-4718 resistance. Silencing of E-cadherin in Merlin-negative PM cells sensitized these cells to VS-4718, strengthening the notion that expression of E-cadherin is a biomarker for indicating resistance to FAK inhibitors [71].

The role of Na (+), K (+), 2Cl (−) cotransport (NLCC1) in early-phase cisplatin-induced morphological and acquired resistance to cisplatin was investigated by comparing PM cell line p31 to a subline, p31 res1.2, using a time-resolved approach. The authors of this study found that the development of cisplatin resistance in P31 cells led to decreased NLCC1 activity, interrupted K+ regulation, and increased basal caspase-3 activity. However, the apoptosis rates in both cell lines were similar when exposed to equitoxic concentrations of cisplatin. The inhibition of NLCC1 activity with bumetanide in P31 cells repressed cisplatin-mediated early-phase membrane bebbling without increasing cisplatin resistance, indicating that active NLCC1 was essential for cisplatin-induced early membrane bebbling but not for resistance to cisplatin [72].

Endoplasmic reticulum (ER) stress occurs when the ER, an organelle responsible for protein synthesis and folding, accumulates misfolded or unfolded proteins, impairing its normal function. This condition induces a cellular response known as the unfolded protein response (UPR), which aims to restore ER homeostasis. Depending on the degree of stress and the cell’s ability to manage it, the UPR can regulate processes such as apoptosis and cell survival [73]. The crucial role of ER stress and UPR mediating chemoresistance in PM cell lines has been reported. In this study, we found that chemoresistant PM cells exhibited deregulated UPR activity, enabling them to survive chemotherapy-induced ER stress. The proteasome inhibitor bortezomib, and FDA-approved drug, selectively targets chemotherapy-recalcitrant PM cells by amplifying UPR signaling, making them susceptible to ER stress-mediated apoptosis. Mechanistically, under ER stress, the protein kinase RNA-like endoplasmic reticulum kinase (PERK) pathway is activated, promoting phosphorylation of elf2α, which augments the translation of activating transcription factor 4 (ATF4). ATF4, in turn, induces the expression of the pro-apoptotic factor C/EBP homologous protein (CHOP) [74].

Y-box binding protein-1 (YB-1), encoded by the *YBX1* gene, is a multifunctional oncoprotein linked with many hallmarks of cancer, such as invasion and metastasis, hypoxic response, and increased chemoresistance in various cancers [75]. The current study has identified YB-1 as a key player in PM’s aggressive characteristics and resistance to therapy. In this study, silencing of YB-1 using small interfering RNA profoundly decreased tumor growth and sensitized PM cells to cisplatin and radiation treatment, suggesting its implication in mediating resistance to such treatments. However, the mechanism by which YB-1 confers resistance to cisplatin was not investigated. Instead, this work emphasizes YB-1’s potential for enhancing PM cells’ sensitivity to cisplatin and radiation [76].

### 2.3. Chemoresistance-Associated miRNAs

MicroRNAs are short non-coding RNAs consisting of 19–25 nt that can regulate gene expression through silencing or modulation of mRNA targets [77]. Through this ability, miRNAs can evoke specific events in regulating the expression of multiple genes, and cellular and molecular components of signaling circuits, affecting the response of cells to chemotherapeutics [78].

MicroRNA-31 is hypothesized to influence chemosensitivity in PM. For instance, the re-expression of miRNA-31 into miRNA-31-null NCl-H2452 cells profoundly enhanced clonogenic resistance to cisplatin and carboplatin. In this investigation, indirect miRNA-31-mediated upregulation of ABCB9, an MDR transporter, was found to be associated with drug accumulation in lysosomes, and increased uptake of platinum by lysosomes. Intriguingly, when miRNA-31 is directly overexpressed, ABCB9 induces cellular chemosensitivity, indicating that miRNA-31 conveys chemoresistance for the most part via an ABCB9-independent mechanism. These data affirm that miRNA-31 loss from PM tumors could promote chemosensitivity and is therapeutically beneficial [79].

Interaction between the miR-379/411 cluster and IL-18 was shown by Yamamoto and colleagues [80]. Using pathway analysis, IL-8 was found to be implicated in the drug sensitivity of PM cells, particularly the correlation between the overexpression of IL-8 and drug resistance. This work also affirmed that the miR-379/411 cluster could promote invasion and induce resistance to pemetrexed and vorinostat (SAHA) by directly targeting IL-8 in PM cells.

MicroRNA-18a is upregulated in PM cell lines [81] and is considered an onco-miRNA in different tumors including NSCLC [82]. Supporting this, Suzuki and colleagues [83] found that mesothelioma cells transfected with the miR-18a inhibitor demonstrated decreased cell proliferation and migration rates compared with non-transfected cells. A chemosensitivity assay revealed that transfection of the miR-18a inhibitor profoundly increased the sensitivity of PM cells to cisplatin but not to pemetrexed.

The presence of miR-186-5p has been linked to drug resistance in treatments such as cisplatin, methotrexate, and taxol by targeting twist-related protein-1 (TWIST-1), a major EMT-related transcription factor in breast cancer [84] and gastric cancer [85,86]. TWIST-1’s role in influencing EMT and metabolic programming may contribute to promoting a chemoresistant phenotype. In an interesting study by Cioce and colleagues [87], the authors found that naturally occurring butein (3,4,2′,4′-tetrahydroxychalcone) exhibits chemosensitizing properties by impinging the miR-186-5p-dependent modulation of TWIST-1. This modulation of TWIST-1 reduced tumor sphere growth, cisplatin resistance, invasion and bioenergetics in the tested PM cells.

Multidrug resistance (MDR) occurs due to an increased efflux of chemotherapeutic drugs, reducing their absorption by cancer cells. One of the known mechanisms of MDR is the regulation of P-glycoprotein (P-gp) [88,89]. miRNA-149 has been identified as a contributory factor to the progression of MDR in taxane-resistant malignant mesothelioma cells by regulating P-gp expression [90]. However, immunohistochemical analysis of P-gp and the expression of multidrug resistance proteins 1 and 2 (MRP1 and MRP2) in PM tissues demonstrated no correlation with patient survival. This indicates that the fundamental resistance of PM is not solely dependent on MDR/P-gp expression or function [91].

Dysregulation of miRNA levels is commonly associated with drug resistance in PM. Downregulated expression of miR-15a, miR-16, and miR-34a has been observed in PM cell lines with acquired drug resistance to cisplatin, gemcitabine, and vinorelbine. Transfection with miR-15a or miR-16 reversed resistance to all three drugs, while miR-34a only reversed resistance to cisplatin and vinorelbine. Mechanistic studies showed that increasing the levels of these miRNAs enhanced drug-induced apoptosis and reduced B-cell lymphoma 2 (BCL2) mRNA and protein levels. Notably, silencing of BCL2 alone was less effective in improving chemosensitivity compared to increasing the miRNA levels. Thus, drug resistance in PM cell lines is linked to the suppression of miR-15a, miR-16, and miR-34a. Boosting their levels sensitizes cells with both acquired and intrinsic resistance, partly through the regulation of BCLS protein expression [92].

The expression of miR-186-5p has been linked to resistance to therapies involving cisplatin, paclitaxel, taxol, and methotrexate [86,93,94,95]. Functionally, miR-186-5p targets twist-related-protein-1 (TWIST1), an EMT-related transcription factor [84], and activates metabolic reprogramming, which may consolidate to the acquisition of a chemoresistance phenotype [33,96]. A recent study has shown that butein, a chemosensitizing agent, suppresses the protumorigenic attributes of PM, at least in part, through the miR-186-5p-TWIST1 axis. At the cellular level, butein triggers miR-186-5p-induced modulation of TWIST1 expression, thereby inhibiting the growth of tumor spheroids, cell invasion, bioenergetics, and cisplatin resistance in the three PM cell lines [87].

### 2.4. Cancer Stem Cell-Induced Chemoresistance

Primary cancer cells originate from stem cells and include a subpopulation known as cancer stem cells (CSCs), which are believed to play a crucial role in drug resistance [97]. One of the basic attributes of CSCs is the ability to self-renew and differentiate into a phenotypically heterogeneous cancer cell population. These cells often remain in a dormant state, making them inherently tolerant to drug treatment [98]. Common chemotherapeutics are generally effective against proliferating cancer cells but fail to target quiescent CSC fractions, leading to increased invasiveness and chemoresistance [99,100]. The drug resistance elicited by CSCs results from a combination of intrinsic and extrinsic factors, involving various biological components and complex mechanisms [101].

In PM research, studies have identified the presence of putative CSCs in various frequencies using established CSC-associated genes and biomarkers including side population (SP), CD9, CD24, CD26, ABCG2, OCT4, BMI-1, uPAR, ALDH, and CD44, strengthening the presence of this cell fraction and its critical role in modulating drug resistance, among others in this malignancy [102,103,104,105,106,107,108,109,110].

Cancer stem cells (CSCs) are resistant to standard and targeted therapies due to several factors, including their ability to repopulate after chemotherapy. Wu and colleagues [111] found that treating murine mesothelioma AB12 cells with cisplatin only temporarily inhibited cancer growth as tumors rapidly reappeared because of cancer cell repopulation between chemotherapy cycles. After chemoradiation, the levels of CSC-associated genes including CD24, CD133, CD90, and uPAR increased significantly, along with mesothelioma stem cell (MSC)-specific genes such as *Tnfsf18*, *Serpinb9b*, *Ly6a*, and *Nppb*. This supports the idea that the stemness properties of MSCs contribute to resistance to chemoradiation.

Consistent with the above findings, our group identified a putative CSC population in PM cell lines characterized by an ALDH^+^/CD44^+^ phenotype and sphere-forming ability. In this case, cisplatin treatment failed to reduce ALDH activity and only temporarily inhibited PM sphere growth. At the transcript level, the cisplatin-resistant cells exhibited upregulated levels of established CSC markers ALDH1A2, ALDH1A3, and CD44, indicating a drug-resistant CSC population [107]. In a separate study, we found that PM cell lines expressed CSC-associated markers BMI-1, uPAR, and ABCG2, unlike normal mesothelial cells. These PM cells showed resistance to cisplatin and pemetrexed accompanied by upregulated mRNA levels of CSC-associated genes *Bm1-1*, *uPAR*, and *ABCG2*, indicating the distinct role of CSCs in drug tolerance [106].

The drug resistance of PM-initiating cells (ICs) to cisplatin and PMX is attributed to the upregulated expression of the drug efflux transporter and multidrug resistance mediator ATP binding cassette subfamily B member 5 (ABCB5). Supporting this, silencing of ABCG5 resensitized IC cells both in vitro and in patient-derived xenograft models. Mechanistically, ABCB5 was transcriptionally activated by the Wnt/GSK3β/β-catenin/c-myc axis, which also enhances the generation of IL-8 and IL-1β. Knocking out IL-8 and IL-1β in IC clones reduced the c-myc-mediated transcription of ABCB5, thereby restoring chemosensitivity. This suggests that Wnt/IL-8/IL-1ß autocrine networks regulate chemoresistance in PM-ICs by upregulating ABCB5 [112].

Transglutaminase 2 (TG2) is a GTP-binding regulatory protein and a key factor in CSC survival and drug resistance. TG2 is highly expressed in PM tumors and mesothelioma CSCs, with them maintaining their CSC phenotype and survival. In vitro experiments showed that silencing TG2 or using a TG2 inhibitor reduced the stemness properties of mesothelioma CSCs, the ability to form tumor spheres, invade Matrigel, and form tumors [113].

The tumor hypoxic microenvironment regulates the resistance of CSCs to chemo- and radiotherapy [114]. In PM-derived spheroids, hypoxic conditions upregulated hypoxia-inducible factor 1 (HIF1a) and HIF2a, including overexpression of its target, Glut-1. The CSC-associated markers OCT4 and CD44 also increased in abundance under hypoxic conditions, leading to enhanced resistance to cisplatin, increased cell mobility and invasiveness of PM cells, and promotion of EMT [115]. Similarly, another study found that the hypoxia adaptation of PM-derived spheres significantly increased resistance to topotecan and pemetrexed. These tumor spheres exhibited high RNA levels of CSC markers CD26, CD44, and ABCG2 as well as hypoxia adaptation markers ABCG2, ALDH1, and HIFs [116].

## 3. Conclusions

**Figure 1:**
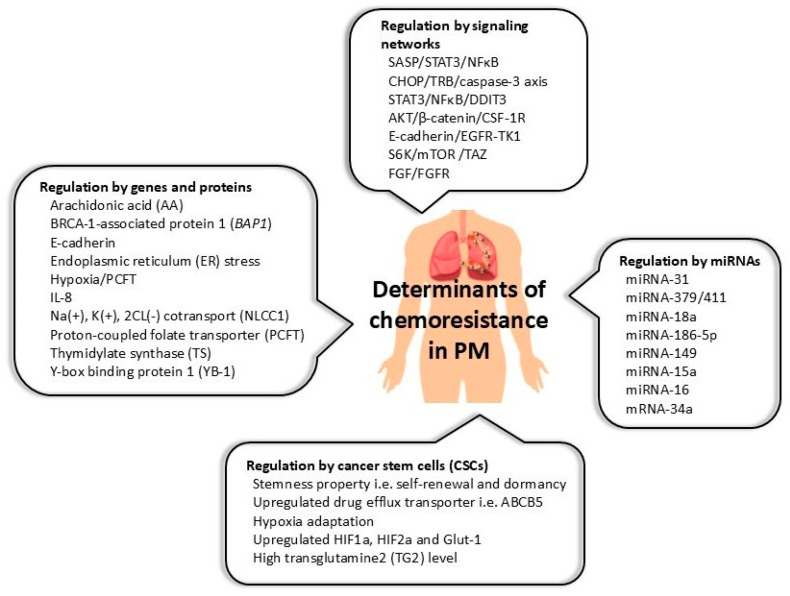
An overview of the different biological determinants and signaling networks classified into four broad categories, regulating the mechanisms of drug resistance in PM. Abbreviations. PM, malignant pleural mesothelioma; ABCB5, ATP-binding cassette subfamily B member 5; CSF-1R, colony stimulating factor-1 receptor; HIF1a, hypoxia-inducible factor 1 subunit alpha; HIF2a, hypoxia-inducible factor 2 subunit alpha; SASP, senescence-associated secretory phenotype, STAT3, signal transducer and activator of transcription 3; NFκB, nuclear factor k-light-chain-enhancer of activated B cells; CHOP, CCAAT-enhancer-binding protein homologous protein; AKT, serine–threonine kinase, also known as protein kinase B (PKB); EGFR, epidermal growth factor receptor; TK1, thymidine kinase; S6K, ribosomal S6 kinase; mTOR, mammalian target of rapamycin; Glut-1, glucose transporter 1; DDIT3, DNA damage-inducible transcript 3; miRNA, micro RNA; YAP, yes-associated protein; TAZ, transcriptional coactivator with PDZ-binding motif; FGR, fibroblast growth factor; FGFR, fibroblast growth factor receptor.

The mechanisms driving drug resistance, representing a crucial problem in PM therapy, are still largely undetermined. This review covers a description of potential determinants including the underlying mechanisms in ensuing treatment recalcitrance in this disease. A substantial body of evidence reveals a complex network of biological compounds and signaling pathways operating at the cellular and molecular levels. The identification of specific tumor resistance-associated proteins, genes, miRNAs, and downstream molecules of signaling circuits and the existence of cancer stem cells (Figure 1) offer promising treatment options for this malignancy, particularly in the development of new drugs in response to known mechanisms of drug resistance.

The paradigm of producing innovative drugs based on specific determinants may further advance a patient-specific treatment approach. Considering the current state of conventional therapeutic modalities (platinum–pemetrexed doublet therapy) [3,117] available for this malignancy and the lack of standard second-line options, patient-tailored combination strategies using innovatively developed drugs, in addition to conventional treatment, may provide brighter prospects. This approach could significantly improve survival rates and quality of life for PM patients.
